# RESEARCH (Recognized effect of Statin and ezetimibe therapy for achieving LDL-C Goal), a randomized, doctor-oriented, multicenter trial to compare the effects of higher-dose statin versus ezetimibe-plus-statin on the serum LDL-C concentration of Japanese type-2 diabetes patients design and rationale

**DOI:** 10.1186/1476-511X-12-142

**Published:** 2013-10-05

**Authors:** Takeshi Inazawa, Kentaro Sakamoto, Takahide Kohro, Raisuke Iijima, Toru Kitazawa, Tsutomu Hirano, Mitsunobu Kawamura, Motoki Tagami, Akira Tanaka, Yasumichi Mori, Tsutomu Yamazaki, Teruo Shiba

**Affiliations:** 1 Kashiwa City Hospital, Chiba, Japan; 2 Toho University Ohashi Medical center, Tokyo, Japan; 3 Jichi Medical University, Tochigi, Japan; 4 Japanese Red Cross Medical Center, Tokyo, Japan; 5 Showa University School of Medicine, Tokyo, Japan; 6 Tokyo Teishin Hospital, Tokyo, Japan; 7 Sanraku Hospital, Life-style related Disease Clinic, Tokyo, Japan; 8 Kagawa Nutrition University, Nutrition Clinic, Tokyo, Japan; 9 Toranomon Hospital, Tokyo, Japan; 10 The University of Tokyo Hospital, Tokyo, Japan; 11 Mitsui Memorial Hospital, Tokyo, Japan

**Keywords:** Hypercholesterolemia, Ezetimibe, Statin

## Abstract

**Aims:**

Hypercholesterolemia coexisting with diabetes still requires clinical intervention to manage the high risk of cardiovascular disease it poses. No second-step strategy is established, however, for cases where strong statins fail to bring cholesterol down to target levels. In this study we seek to demonstrate the superior effect of ezetimibe in combination with strong statins to reduce LDL-C in Japanese patients suffering from both T2DM and hyper LDL-cholesterolemia.

**Methods:**

T2DM outpatients (109 patients from 16 institutes) who failed to achieve the target LDL-C value were recruited and randomly assigned to two groups, a double-dose-statin group and ezetimibe-plus-statin group. Follow-ups were scheduled at 0, 12, 26, and 52 weeks. The primary endpoint was the percentage change in the level of LDL-C from baseline to 12 weeks.

**Interim results:**

We could successfully create randomized (gender, age, LDL-C, HbA1c, etc.) two groups except for slight differences in apolipoprotein-B and sd-LDL.

**Conclusions:**

RESEARCH is the first prospective, parallel-group, multicenter study comparing a double dose of strong statin with ezetimibe plus strong statin for T2DM patients. The RESEARCH study will provide reliable evidence with which to establish a clinical strategy for diabetics who fail to achieve the target LDL-C value.

## Background

The incidence of metabolic disease, a condition often associated with coronary artery disease (CAD) or stroke is increasing substantially all over the world. According to a Japanese government publication from 2010, some 17.4 million cases of diagnosed or suspected diabetes were reported in the country, about 31.4% more than the number reported in the earlier surveillance from 2002 [1]. Among them, patients with diabetes mellitus face a strong risk of both CAD and stroke. JDCS [2], a cohort study of Japanese type 2 (T2) diabetics, reported a CAD incidence of 9.6/1,000 T2DM patients/year and a stroke incidence of 7.5/1,000 T2DM patients/year. The Hisayama Study [3], another Japanese cohort study, respectively showed CAD and stroke incidences of 8.0 and 10.2 /1000 persons/year in type 2 diabetic patients, versus incidences of 3.4 and 4.0/1,000 persons/year in subjects with normal glucose tolerance. According to those data, Japanese diabetics were exposed to a two-fold higher risk of cardiovascular disease than subjects with normal glucose tolerance.

The risk of cardiovascular disease in diabetes mellitus is pushed sharply higher by hypercholesterolemia, especially elevated low-density lipoprotein cholesterol (LDL-C). Apart from dietary and lifestyle intervention, hydroxylmethylglutarylcoenzyme-A -reductase -inhibitors (statins) have been the primary component of LDL-C-lowering therapy. The MEGA study [4], a randomized controlled trial from Japan, has shown that LDL-C reduction by pravastatin reduces the incidences of CAD and stroke in Japanese subjects, effectively duplicating the results from larger trials with global populations [5-8]. Patients who fail to achieve the LDL-C target with a regular dose of strong statins have the option of increasing the statin dose or combining the statins with other classes of lipid-lowering medications such as ezetimibe, bile acid sequestrans, fibrates, niacins, or ω3-fatty acids. Ezetimibe, a cholesterol absorption inhibitor, targets the cholesterol transporter protein Niemann Pick C1 like 1 at the jejunal enterocyte brush border and localizes there to inhibit the absorption of cholesterol from the gastrointestinal and biliary systems [9-11]. Ezetimibe administered in combination with statin reduces LDL cholesterol levels and the incidences of the following major atherosclerotic events: nonfatal myocardial infarction, coronary death, nonhemorrhagic stroke, and revascularization procedures in CKD [12]. In this study we seek to demonstrate the superior effect of ezetimibe-plus-strong-statin in reducing LDL-C levels in Japanese patients suffering from both type 2 diabetes mellitus (T2DM) and hyper LDL cholesterolemia.

## Methods/design

The RESEARCH study is a prospective, randomized, parallel-group, active-controlled, doctor-oriented, multicenter, clinical trial designed to examine whether ezetimibe combined with a regular dose of strong statin significantly lowers the serum LDL-C concentration in Japanese T2DM patients, compared with a double dose of strong statin administered without ezetimibe.

The study is conducted and managed with the approval of the ethics committee at each institute and conforms closely with the Declaration of Helsinki and the guidelines from the Japanese Ministry of Health, Labor and Welfare (complete revision on December 28, 2004). The protocol has been registered in the UMIN Clinical Trials Registry (UMIN000002593) [13].

### Patients

Entry criteria and exclusion criteria are shown in Table 1. The patients enrolled in this study were T2DM outpatients who had failed to achieve the LDL-C target recommended by the Japanese guideline, namely, LDL-C < 120 mg/dL for patients without CAD or LDL-C < 100 mg/dL for patients with CAD [14], in spite of receiving strong statin treatment at the standard dose according to Japan’s national health insurance policy (10 mg/day of atorvastatin or 1 mg/day of pitavastatin). Changes in LDL-C values were examined using Friedewald’s formula, so patients with hypertriglyceridemia of over 400 mg/dL had to be excluded.

**Table 1:** Entry and exclusion criteria

**Entry criteria**	
Japanesess	
Type 2 diabetes mellitus	
Age ≥ 20 years	
Using atorvastatin 10 mg/day or pitavastatin 1 mg/day for > 1 month	
LDL-C ≥ 120 mg/dL for patients without CAD , LDL-C ≥ 100 mg/dL for patients with CAD	
Exclusion criteria	
Pregnant, possible to become pregnant, or brest-feeding to baby(s)	
Hypersensitive to the medicines concerned	
Liver dysfunction	
HbA1C ≥ 9.4%	
Serum creatinine ≥ 2.0 mg/dL	
Triglyceride ≥ 400 mg/dL	
Secondary dyslipidemia, drug-induced dyslipidemia	
Homozygotic familial hypercholesterolemia	
Other non-specific reason, such as poor adherence	

CAD: coronary artery disease.

Patients who provided informed consent were randomly assigned to two groups, a double-dose-statin (DS) group and ezetimibe-plus-statin (ES) group. The patients were randomized through a system for online central registration. To reduce heterogeneity between groups, dynamic allocation was adopted using age and gender as assignment factors. The results of the randomization were open to both the patients and doctors.

### Treatments and data collection (Figure 1)

**Figure 1:**
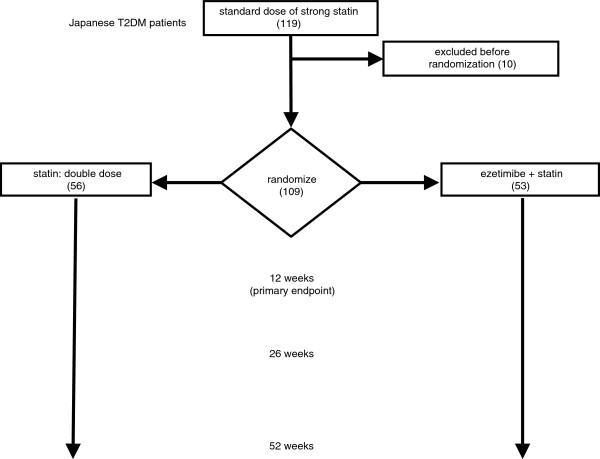
**Design scheme for the RESEARCH study.** The numbers of patients appear inside brackets. T2DM: type 2 diabetes mellitus.

The patients assigned to the DS group are given double the basal dose of statin. The patients assigned to the ES group are given the basal dose of statin in combination with 10 mg/day of ezetimibe. Data are collected at 0, 12, 26, and 52 weeks. After 12 weeks of treatment, a higher dose of statin is allowed, when necessary, in the ES group, and a higher dose of either statin or ezetimibe is allowed, when necessary, in the DS group. The patients receive none of the following agents during the study: statins other than atorvastatin or pitavastatin, anion-exchanging resin agents, fibrates, nicotinic acids, eicosapentaenoic acid, probucol, and other lipid-lowering agents.

### End points

The primary endpoint is the percentage change in the level of serum LDL-C from baseline to week 12 of treatment. Along with the LDL-C decrements, percentage of patients who reached the treatment goals (LDL-C <120 mg/dl or <100 mg/dl) was placed at one of the secondary endpoints. Another major secondary endpoint is the effects to the particle size of LDL, whereby measured by amount of small dense low-density lipoprotein (sd-LDL) or remnant-like particle cholesterol (RLP-C). The other secondary endpoints are the high-density lipoprotein cholesterol (HDL-C), total cholesterol (TC), triglyceride (TG), high sensitivity C-reactive protein (HS-CRP), and HbA1c, as well as the rate at which the subjects achieve the LDL-C target recommended under the Japanese guideline.

Whereas the effect of the modification will be observed about the patients to whom treatment changes will be applied by not achieving the target level during the extended periods after 12 weeks, long term effects of the regimen of each group will be evaluated as to the subjects without treatment modification in the same period.

### Data measurement

Samples for sd-LDL are collected at Professor Tsutomu Hirano’s laboratory (Showa University, Tokyo, Japan) and measured by his precipitation method [15]. Samples for RLP-C and HS-CRP are collected and measured in a single laboratory (SRL Inc. Tokyo, Japan). LDL-C values are determined with the Friedelwald’s formula (LDL-C = TC – HDL-C – TG/5). Non-HDL-C values are calculated as TC – HDL-C. The measurements for TC, HDL-C, and TG and routine laboratory tests for parameters such as HbA1C, glucose, AST, ALT, and CK are performed at the participating institutes. Malondialdehyde modified LDL (MDA-LDL) is measured only for patients with CAD, in order to stay within the restrictions set under Japan’s national health insurance policy.

### Study organization

The contributors to this study are listed in Table 2. The chair investigator is Professor Teruo Shiba at Toho University Ohashi Medical Center. Study coordination and monitoring are consigned to Nouvelle Plus Co., Ltd. of Tokyo, Japan.

**Table 2:** Members of the RESEARCH Study Group

**Investigator**	**Institute**
Teruo Shiba	Toho University Ohashi Medical Center
	Mitsui Memorial Hospital
Akira Tanaka	Kagawa Nutrition University, Nutrition Clinic
Tsutomu Hirano	Showa University School of Medicine
Tsutomu Yamazaki	The University of Tokyo Hospital
Mitsunobu Kawamura	Tokyo Teishin Hospital
Takahide Kohro	Graduate School of Medicine, The University of Tokyo
Takeshi Inazawa	Kashiwa City Hospital
Motoki Tagami	Sanraku Hospital, Life-style related Disease Clinic
Masao Omura	Yokohama Rosai Hospital
Motoyoshi Tsujino	Tokyo Metropolitan Tama Medical Center
Takayuki Watanabe	Yokohama City Minato Red Cross Hospital
Toshiyuki Horiuchi	Toshima Hospital
Toru Hiyoshi	Japanese Red Cross Medical Center
Nobuo Sekine	Tokyo Kosei Nenkin Hospital
Rina Chin	Tokyo Kyosai Hospital
Keiko Ashidate	Kudanzaka Hospital
Yasumichi Mori	Toranomon Hospital

### Statistical design and analysis

Under the current assumptions, 60 patients need to be enrolled in each group. This number is estimated from the following hypothesis: a doubled statin dose leads to a 11.7% reduction in LDL-C, whereas the addition of ezetimibe leads to a 21.7% reduction in LDL-C (standard deviation: 15%). The significance level is set at 5%, power is set to 90%, and the follow-up ratio is estimated to be 80%.

About the end points, differences between the two groups for categorical variables will be assessed using the χ^2^-test, and continuous variables will be evaluated using the Wilcoxon test. The Wilcoxon signed-rank test will be used to assess the difference in parameters before and after treatment within each group. Significance is set at a p value of less than 0.05 (2-sided) in all analyses. All data will be analyzed using Stata® version 12.1 (StataCorp LP., Texas, USA).

## Interim results

Informed consent was obtained from 119 patients at 16 institutes in Japan over the period from July 2009 to June 2012. The recruiting period was initially set at 2 years, but extended to 3 years based on the progress of subject registration. After excluding 10 patients who ceased to conform with the entry criteria or who canceled their consent, 109 patients were randomly assigned to two treatment groups: the DS group (n = 56) and the ES group (n = 53).

The profile of basal treatment was as follows: none (n = 24), oral treatment (n = 58), injection treatment (n = 27) including single treatment of insulin (n = 9) and GLP-1 receptor agonist (n = 2). Totally 74 patients was taking some sort of oral Antidiabetic agents. The number of patients taking each class of agents was 19 for thiazolidinedione (TZD), 36 for sulfonylurea (SU), 40 for biguanide (BG), 21 for α-glucosidase inhibitor (α-GI), 26 for dipeptidyl peptidase IV (DPP-4) inhibitors, and 7 for glinides.

There were no significant differences between the two groups in gender or in the percentages of subjects with a smoking habit, hypertension, hypertriglyceridemia, hypo-HDL-cholesterolemia, chronic kidney disease, history of stroke, history of CAD, or family history of CAD (Table 3). Likewise, there were no significant differences between the groups in age or the levels of TC, TG, HDL-C, LDL-C, non-HDL-C, RLP-C, MDA-LDL, apolipoprotein (apo)-A1, apo-E, fasting glucose, fasting insulin, ALT, AST, γ-GTP, CK, creatinine, HbA1C, or HS-CRP. There were significant but slight differences between the groups in the values of apo-B and sd-LDL (Table 4). We think the randomization was quite successful. As the participant institutes mainly engage in outpatient care for diabetes, subjects with a history of cardiovascular disease were rather rare. The HbA1c value (7.20% in total) was not excellent, but slightly better than that in JDCS [2].

**Table 3:** Patient backgrounds, categorical variables

**Number of cases**		**Double dose of statin**	**Statin + ezetimibe**	**p value**
		**n = 56**	**n = 53**	
Male	%	57.1	58.5	0.887
Smoking	%	23.6	25.0	0.869
Family history of CAD	%	9.3	9.6	0.950
Hypertension	%	67.9	58.5	0.311
Hypertriglyceridemia	%	34.5	41.5	0.456
CKD	%	16.1	9.4	0.301
Low HDL-C	%	3.6	7.5	0.363
History of stroke	%	3.6	0.0	0.169
History of CAD	%	10.7	15.1	0.495

The data were assessed using the χ^2^-test. CAD: coronary artery disease.

**Table 4:** Baseline data on the patients, continuous variables

	**Double dose of statin**		**Statin + ezetimibe**		**p value**
	**mean ± sd**	**n**	**mean ± sd**	**n**	
age	62.6 ± 9.5	56	61.7 ± 11.1	53	0.4949
TC	218.2 ± 27.2	56	212.5 ± 30.1	53	0.3567
TG	161.9 ± 88.3	56	146.7 ± 95.2	53	0.1803
HDL-C	54.7 ± 9.6	56	56.7 ± 15.2	53	0.9734
LDL-C	135.2 ± 22.6	56	130.6 ± 19.2	53	0.5463
non HDL-C	163.6 ± 25.7	56	155.9 ± 26.9	52	0.1133
RLP-C	6.3 ± 3.9	56	6.1 ± 4.8	52	0.4756
apo A1	146.6 ± 16.6	44	144.2 ± 19.2	40	0.3288
apo B	111.4 ± 18.2	44	101.8 ± 16.5	40	0.0152
apo E	4.1 ± 0.9	44	3.8 ± 1.0	40	0.1604
MDA-LDL	157.0 ± 62.0	12	112.6 ± 27.7	9	0.0549
sd-LDL	52.2 ± 17.9	56	46.2 ± 16.0	52	0.0484
Fasting glucose	132.7 ± 32.5	56	141.5 ± 33.1	53	0.1108
Fasting insulin	10.9 ± 7.0	35	13.8 ± 12.5	28	0.5381
ALT	23.6 ± 10.9	56	24.7 ± 12.5	53	0.8318
AST	23.1 ± 12.1	52	23.7 ± 12.2	49	0.8541
γ-GTP	30.2 ± 21.7	53	28.6 ± 26.6	47	0.3248
CK	116.7 ± 58.2	54	122.5 ± 88.3	51	0.9642
Creatinine	0.81 ± 0.28	56	0.79 ± 0.24	51	0.9627
HbA1C	7.15 ± 1.32	56	7.25 ± 0.64	53	0.6294
hs-CRP	1114 ± 1493	56	922 ± 1119	51	0.6025

The data were evaluated using the Wilcoxon test.

## Discussion

When a patient on statins fails to attain good control of LDL-C, a doubling of the statin dose or the addition of ezetimibe seems to be strong choice for general practitioners. Compared to statins alone, the co-administration of ezetimibe with statins has previously been shown to produce significant incremental reductions in the plasma levels of LDL-C in the general population [16-19]. Since diabetics with hyper-LDL-cholesterolemia are shown to be at high risk for CV events [20] and only around half of diabetic patients meet the LDL-C goal both in the U.S. [21] and in Japan [22], we focused on T2DM patients who were unable to attain the target LDL-C values proposed under the Japanese guideline even when receiving regular doses of strong statins in accordance with the recommendations under Japan’s national health insurance policy. We are thus comparing the LDL-C-lowering effect of a double dose of a strong statin and a regular dose of a strong statin plus ezetimibe in diabetic patients.

Simultaneously, we focus on the particle size changes of LDL-C that may reflect the difference in the mechanisms of these agents which reduce amount of LDL-C. Especially, small-dense low-density lipoprotein (sd-LDL) is recently recognized to be an independent cardiovascular risk marker, presenting as the change in LDL particle size. We evaluated the difference of these two treatments via the major secondary endpoint.

Two earlier reports have compared the effects of co-administration of ezetimibe with a high dose of a standard statin (20 mg of simvastatin plus 10 mg of ezetimibe) with the effects of a double dose of a prescribed standard statin (40 mg of simvastatin in T2DM patients who had been treated with 20 mg of simvastatin). In both of these studies, the mean percent reductions in LDL-C from baseline were significantly greater in the ezetimibe-plus-simvastatin group than in the double-dose simvastatin group [23,24]. In RESEARCH we are looking at the effect of add-on ezetimibe with a strong statin compared to the effect of a double dose of a strong statin. We are also looking at ethnic differences among diabetic patients to analyze whether differences in dietary habits, lifestyles, or physiological reactions influence the atherogenic properties of serum lipid profiles. The clinical backgrounds of the diabetic patients in the earlier studies have also differed. One study followed diabetic patients treated with thiazolidinediones [23], while the other enrolled only diabetic patients with a history of CAD [25]. Another study compared 80 mg of atorvastatin versus 40 mg of atorvastatin plus 10 mg of ezetimibe in T2DM patients who had been treated with 40 mg of atorvastatin alone [25]. In that study, the mean percent reduction in LDL-C from baseline after 6 weeks of treatment was greater in the atorvastatin-plus-ezetimibe group (27%) than in the double-atorvastatin group (10%). Like us they used a strong statin, but unlike us they used atorvastatin, administered at super-high doses, and enrolled patients of variable races. Additionally, their patients were T2DM, metabolic syndrome without T2DM, or subjects with neither metabolic syndrome nor T2DM, hence their analysis on T2DM was *post hoc* and insufficient to show statistical significance. Our study is prospective and our targets are exclusively T2DM patients on a regular course of strong statins who nonetheless have failed to achieve the LDL-C target recommended under the Japanese guideline. It remains to be seen whether a double dose of strong statins in those patients is more effective than a regular dose of strong statins with ezetimibe, or vice versa. Few reports have addressed this controversial issue, especially in Japan. According to a single center, crossover study from Japan on type-2 diabetes patients or IGT (impaired glucose tolerance) with CAD, co-administration of ezetimibe was superior to a double dose of strong atorvastatin [26]. Yet in another report from Japan described in Japanese literature, treatment with ezetimibe plus pitavastatin brought about more or less the same LDL-C reduction as a double dose of pitavastatin.

We can reasonably hypothesize that a dual therapy of ezetimibe and statins has an edge in lowering LDL-C in T2DM patients, given that these agents affect serum LDL-C level through different mechanisms, one via reduction of intestinal cholesterol absorption, the other via a mighty inhibition of hepatic cholesterol synthesis. It would also be of clinical value to assess how the co-administration of ezetimibe with statins affects the atherogenic lipid profile in diabetic patients. None of the abovementioned studies assessed the effect on sd-LDL, a strong risk factor for CAD [15,27], or on RLP-C, an independent cardiovascular disease risk factor [28]. Ezetimibe therapy is reported to reduce RLP-C in Japanese patients with hyper-LDL-cholesterolemia [29]. Ezetimibe affects the size of LDL particles and reduces the concentration of sd-LDL in T2DM patients [30]. Studies comparing the effects of these two treatments collaterally on the apolipoproteins revealed no differences in the effects on apo A1, apo C3, or apo E, whereas co-administration of ezetimibe with statins significantly decreased apo B, the apo found in non-HDL lipoproteins [19,25].

Our study will be the first prospective, parallel-group, multicenter comparison between a double dose of a strong statin and the combination of ezetimibe plus a strong statin for T2DM patients in actual outpatient clinics (i.e., patients receiving various treatments for glycemic control) who take strong statins but nonetheless fail to achieve the LDL-C target recommended under the Japanese guideline. The patients receiving ezetimibe–plus-statin regimen are expected to show significant reductions in the levels of LDL-C, Non-HDL-C, sd-LDL, and apo-B, and to achieve the LDL-C target recommended under the guideline at a high rate. The RESEARCH study is a necessary step to collect reliable evidence for diabetologists, cardiologists, and general practitioners.

## Competing interests

The other authors declare they have no competing interests.

## Authors’ contributions

TS is responsible for overall design and registry. TI is assigned to the study design and writing this paper. MK, MT, AT, and YM also contribute to the study design. KS, RI, TK contribute to the study practice including the recruitment of the subjects. TH is in charge of the measurement of sd-LDL. TK and TY are involved in data analysis. All authors read and approved the final manuscript.
